# Patient self-appraisal of change and minimal clinically important difference on the European organization for the research and treatment of cancer quality of life questionnaire core 30 before and during cancer therapy

**DOI:** 10.1186/1471-2407-13-165

**Published:** 2013-03-28

**Authors:** Fanxing Hong, Jaclyn L F Bosco, Nigel Bush, Donna L Berry

**Affiliations:** 1 Department of Biostatistics and Computational Biology, Dana-Farber Cancer Institute, Harvard School of Public Health, Boston, MA, USA; 2 Phyllis F. Cantor Center, Dana-Farber Cancer Institute, Department of Medicine, Harvard Medical School, Boston, MA, USA; 3 National Center for Telehealth and Technology, Joint Base Lewis-McChord, Tacoma, WA, USA

**Keywords:** Cancer treatment, Health related quality of life, Quality of life questionnaire-core, Subject significance questionnaire, Minimal clinically important differences

## Abstract

**Background:**

Clinical interpretation of health related quality of life (HRQOL) scores is challenging. The purpose of this analysis was to interpret score changes and identify minimal clinically important differences (MCID) on the European Organization for Research and Treatment of Cancer Quality of Life Questionnaire-Core 30 (QLQ-C30) before (T1) and during (T2) cancer treatment.

**Methods:**

Patients (N = 627) in stem cell transplant (SCT) and medical (MED) or radiation (RAD) oncology at two comprehensive cancer centers, enrolled in the Electronic Self-Report Assessment-Cancer study and completed the QLQ-C30 at T1 and T2. Perceived changes in five QOL domains, physical (PF), emotional (EF), social (SF), cognitive functioning (CF) and global quality of life (QOL), were reported using the Subject Significance Questionnaire (SSQ) at T2. Anchored on SSQ ratings indicating “improvement”, “the same”, or “deterioration”, means and effect sizes were calculated for QLQ-C30 score changes. MCID was calculated as the mean difference in QLQ-C30 score changes reflecting one category change on SSQ rating, using a two-piece linear regression model.

**Results:**

A majority of SCT patients (54%) perceived deteriorating global HRQOL versus improvement (17%), while approximately equal proportions of MED/RAD patients perceived improvement (25%) and deterioration (26%). Global QOL decreased 14.2 (SCT) and 2.0 (MED/RAD) units, respectively, among patients reporting “the same” in the SSQ. The MCID ranged 5.7-11.4 (SCT) and 7.2-11.8 (MED/RAD) units among patients reporting deteriorated HRQOL; ranged 2.7-3.4 units among MED/RAD patients reporting improvement. Excepting for the global QOL (MCID =6.9), no meaningful MCID was identified among SCT patients reporting improvement.

**Conclusions:**

Cancer treatment has greater impact on HRQOL among SCT patients than MED/RAD patients. The MCID for QLQ-C30 score change differed across domains, and differed for perceived improvement and deterioration, suggesting different standards for self-evaluating changes in HRQOL during cancer treatment. Specifically, clinical attention can be focused on patients who report at least a 6 point decrease, and for patients who report at least a 3 point increase on QLQ-C30 domains.

**Trial registration:**

The trial was registered with ClinicalTrials.gov: NCT00852852

## Background

Health-related quality of life (HRQOL) is an important patient outcome measure following cancer treatment in randomized trials. HRQOL was shown to be an independent prognostic factor for response to treatment, progression-free survival, and survival [1,2]. Significance of differences (or changes) in HRQOL are often interpreted with statistical hypothesis testing using p-values [3]. However, a statistically significant difference is not synonymous with clinical meaningfulness. Clinical investigators are challenged to interpret important changes in HRQOL over time and to determine a minimal clinically important difference (MCID). Once established, a MCID is a useful benchmark for clinical researchers to assess effectiveness of an intervention and determine sample sizes for future clinical trials. Understanding the MCID may help clinicians address HRQOL related issues during cancer treatment.

The European Organization for the Research and Treatment of Cancer (EORTC) Quality of Life Questionnaire Core 30 (QLQ-C30) [4] is a commonly used instrument for measuring HRQOL among cancer patients. Osoba et al. evaluated 375 patients with metastatic small cell lung cancer or breast cancer, and observed a mean change of 5–10, 10–20, >20 units for small, moderate, large changes, respectively, in QLQ-C30 scores [5]. In a review of 14 cross-sectional studies, King et al. recommended that a change of 5 and 15 units was a relatively small and large difference, respectively [6]. On the contrary, Grulke and colleagues evaluated trends in HRQOL scores before and after hematopoietic stem cell transplant (SCT) from 33 studies that involved 2,800 patients in England and Germany, and concluded that only a difference exceeding 15 units was clinically significant [7]. Additionally, in a meta-analysis of 152 cross-sectional studies (15% were conducted in the US/Canada regions), Cocks et al. recommended a range of 9 to 19 points as the medium difference [8]. Most of these studies analyzed data among European patients, and focused on patients with specific cancer types. To our knowledge, our analysis is the first to interpret and to identify MCIDs for the QLQ-C30 score changes focusing on American patients with cancer.

There are few analyses assessing potential differences in MCID between improvement and deterioration. Ringash et al [9] and Cella et al [10] analyzed the Functional Assessment of Cancer Therapy (FACT) and reported a larger magnitude in MCID for deterioration than for improvement. This is in contrast to a study using QLQ-C30 among patients treated for brain cancer, in which Maringwa and colleagues suggested no clear indications that the MCID differed between improvement and deterioration [11]. Kvam et al. reported a MCID of 8 and 12 units in QLQ-C30 for improved and deteriorated HRQOL among patients with multiple myeloma [12]. Both of the two studies focused on specific patient population. Using a unique approach of assembling expert opinions, Cocks et al. reported smaller estimates for improvement than for declines in a meta-analysis of 118 published longitudinal studies [13]. It is not yet established whether the different magnitudes of MCID should be used in QLQ-C30 as clinically meaningful benchmark for improvement and deterioration.

One well-accepted definition for MCID is “the smallest difference in score in the domain of interest which patients perceived as beneficial and which would mandate, in the absence of troublesome side effects and excessive cost, a change in the patient’s management” [14], p. 408. Two approaches are commonly used to assess MCID. The distribution-based approach utilizes the statistical features, such as fractions of the standard deviation (SD). The anchor-based approach is preferred because it uses patient-derived ratings rather than statistical significance [5]. In the current analysis, we used an anchored-based approach based on the methodology introduced by Osoba et al. in which patients were asked to rate their perceived change in HRQOL over time using the Subject Significance Questionnaire (SSQ) [15].

The objectives of this analysis were (a) to report and interpret HRQOL change measured by QLQ-C30, and (b) to determine the MCID for the QLQ-C30 change scores over time before and during cancer therapy among American patients with various types of cancer.

## Methods

### Study sample

A total of 765 adult, ambulatory patients with any type of cancer, who started a new medical, radiation or stem-cell transplantation treatment at one of two comprehensive cancer centers (Seattle Cancer Care Alliance or the University Of Washington Medical Center) were enrolled into the Electronic Self-Report Assessment for Cancer (ESRA-C) intervention trial (NCT00852852). The study was approved by the Institutional Review Board of the Fred Hutchinson Cancer Research Center/University of Washington Cancer Consortium. The primary outcome was reported elsewhere [16].

Using touch-screen, notebook computers, patients completed e-versions of the QLQ-C30 pre-treatment (T1) and during treatment (T2). Most of the SCT patients answered the T2 assessment at the first, post-hospital discharge clinic visit. At T2, patients reported perceived changes in quality of life by completing a seven-point response category SSQ. Eighty-six percent (n = 660) completed the T2 assessment. Additional details of the full sample and study procedures have been reported previously [16].

### Analytic variables

Patients reported socidemographic characteristics at enrollment. Information on cancer type and incident or recurrent diagnosis was abstracted from medical records. The QLQ-C30 [4] is a cancer-specific quality of life instrument with five functional subscale scales- physical (PF), role (RF), emotional (EF), social (SF) and cognitive (CF) functioning, plus global QOL. The QLQ-C30 summary scores for each domain were transformed to range from 0 to 100 according to published methods for version 3 [17]. Higher functional and global QOL scores correspond to a higher level of functioning. For the current study, alpha coefficients for the subscales ranged from 0.66 (CF) to 0.87 (global QOL) at T1 and 0.70 (CF) to 0.89 (global QOL) at T2.

The five SSQ items correspond with the QLQ-C30 domains of PF, EF, SF, CF and global QOL. The SSQ queries patients about their perceived level of change in each of the domains using a seven-point scale ranging from (1) very much worse, (2) moderately worse, (3) a little worse, (4) about the same, (5) a little better, (6) moderately better, to (7) very much better. The SSQ instrument has been used as a calibration instrument to assess the magnitude of changes in HRQOL that were perceived and considered meaningful to patients as measured by validated instruments such as the QLQ-C30 [5,15,18]. We analyzed the PF, EF, SF CF, and global QOL domains in comparison to the corresponding SSQ items.

### Statistical analysis

Baseline demographic and clinical characteristics were summarized using descriptive statistics among SCT and MED/RAD patients (Table 1). We used Inter-Quartile Range (IQR) criteria to identify outliers and removed 33 patients with longer than 109 days between T1 and T2 from subsequent analyses. As the result, the final analytic sample contains 627 patients. Due to different patterns of HRQOL change observed over time, patients treated with SCT and in MED/RAD oncology were analyzed separately.

**Table 1:** Demographic and clinical characteristics of 627 medical/radiation (MED/RAD) oncology and transplant (SCT) patients

	**Clinical service**			
	**SCT( n = 191)**		**MED/RAD (n = 436)**	
	**N**	**%**	**N**	**%**
**Sex, Male**	111	58.1	229	52.5
**Age, years**	49 (12.9)		56 (13.9)	
Mean (SD)				
Range	19-75		18-89	
**Ethnicity, Hispanic/Latino**	2	1.0	9	2.1
**Race, minority or multiple**	13	6.8	32	7.3
**Some college or college graduate**	146	76.4	299	68.6
**Married/Partnered**	138	72.3	303	69.5
**Low annual household income (<=$35,000)**	44	23.0	101	23.2
Study Group (Intervention)*	92	48.2	217	49.8
**Incident cancer diagnosis**	134	70.2	370	84.9
**Cancer type**	0	0	42	9.6
Breast				
GI	0	0	80	18.3
GU	0	0	70	16.1
Gyn	0	0	49	11.2
Head and Neck	0	0	53	12.2
Leukemia	88	46.1	6	1.4
Lung	0	0	41	9.4
Lymphoma	62	32.5	40	9.2
Myeloma	38	19.9	7	1.6
Other	3	1.5	48	11.0
**Days between T1 and T2, days**				
Median, range	42 (14–107)		41(11–105)	

*In the intervention group, Patient-reported quality-of-life issues were automatically displayed on a graphical summary and provided to the clinical team before an on-treatment visit; in the control group, no summary was provided. Details on study design can be found in Berry et al. [16].

**Figure 1:**
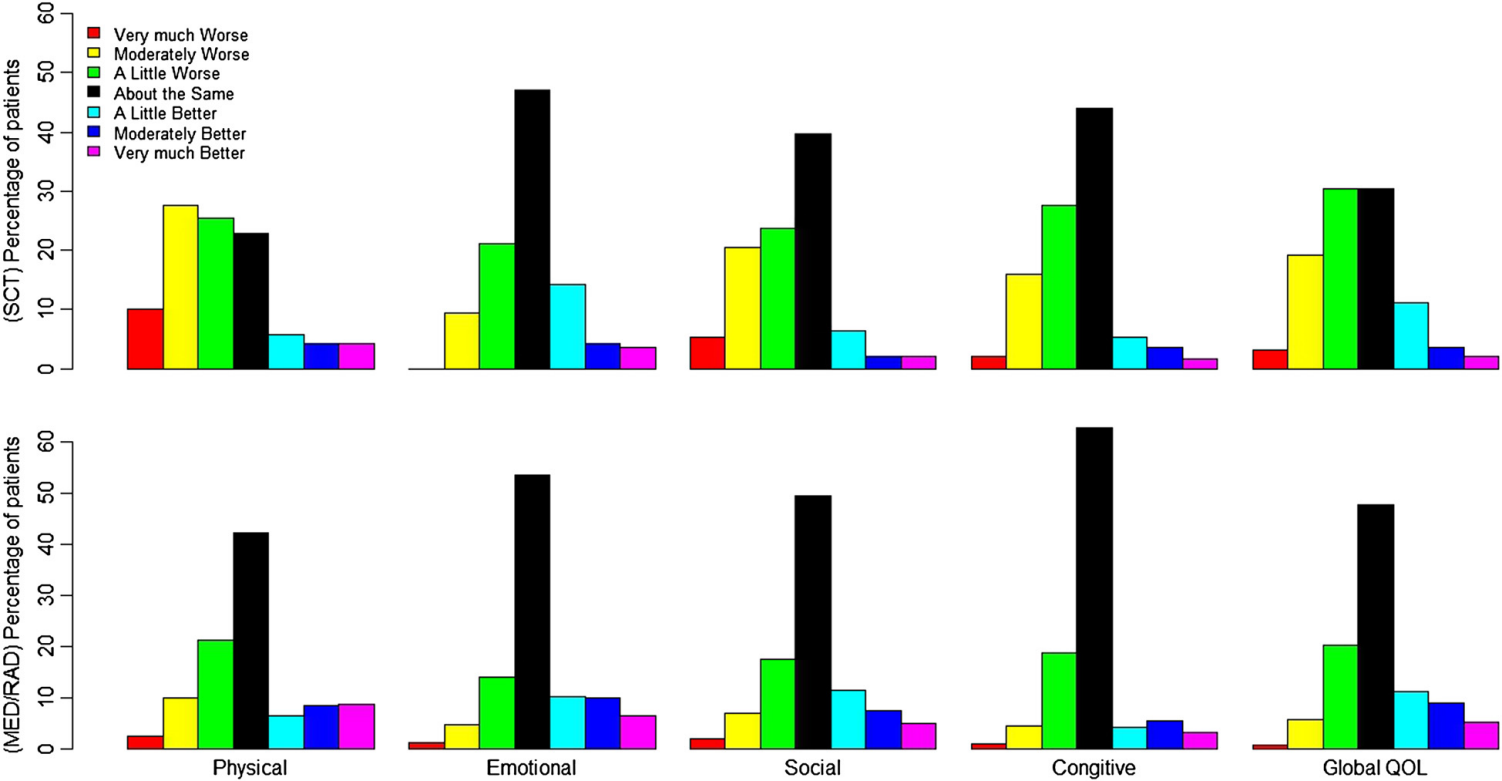
**Percentage of patients reporting SSQ rating of changes, for SCT (top) and MED/RAD oncology (bottom).** Column represents % of patients.

**Figure 2:**
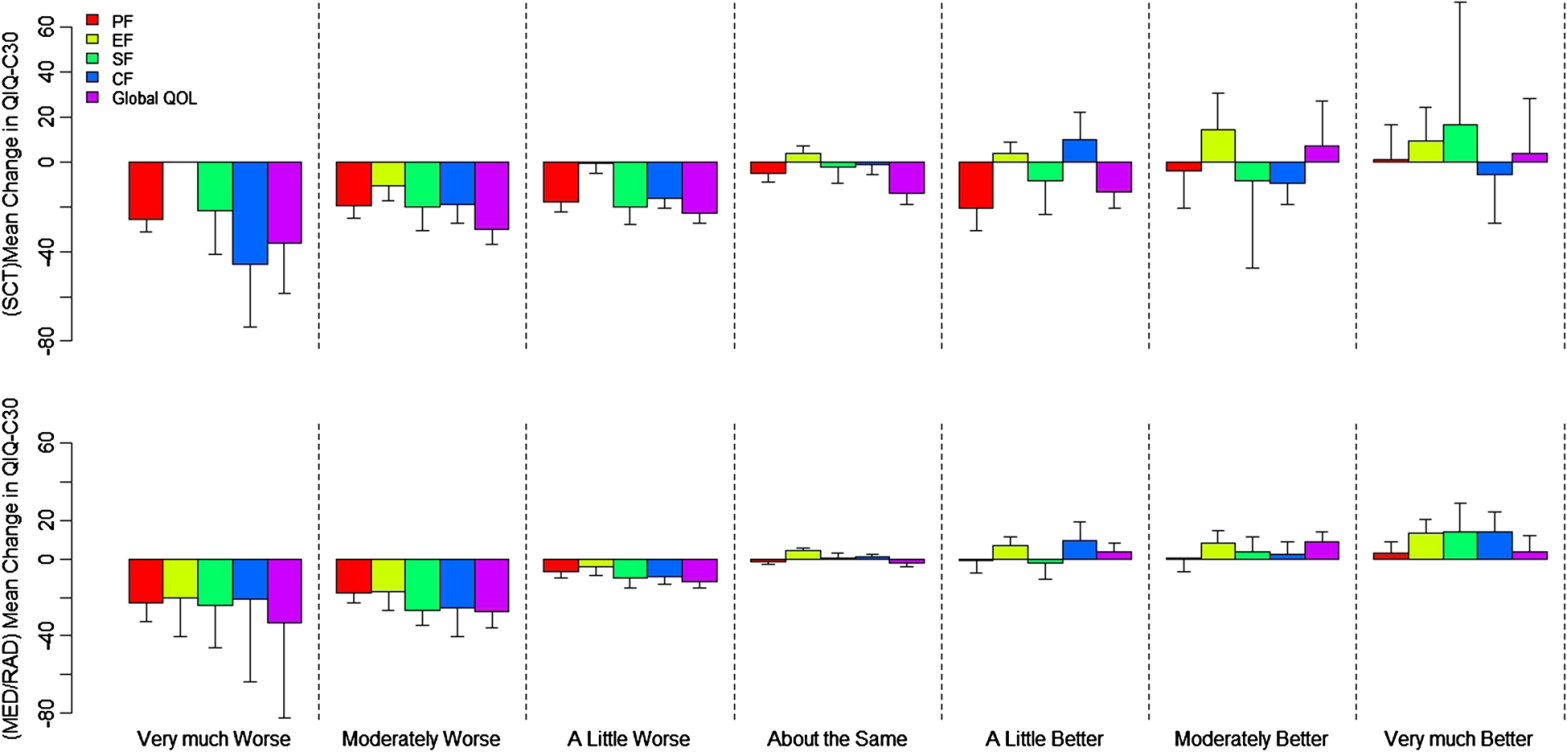
The relationship between mean change in QLQ-C30 score and SSQ rating of change for SCT (top) and MED/RAD oncology (bottom), column represent mean change and 95% CI.

The score change was calculated as the difference in QLQ-C30 between T2 and T1. Nonparametric Spearman rank correlation coefficients were calculated between QLQ-C30 score change and response categories of the SSQ. A matrix of QLQ-C30 domains and SSQ ratings was created (Table 2, Figures 1 and 2). For reporting purposes, we refer to entries in the matrix as “instances.” A total of 35 instances were formed across the five QLQ-C30 domains and seven SSQ rating categories; improvement or deterioration was represented by 15 instances and “the same” involved 5 instances. For each instance, we calculated the mean QLQ-C30 score change and the effect sizes (the mean change score divided the standard deviation). Negative (or positive) values indicated a lower (or higher) QLQ-C30 score at T2, and were considered in the same direction as SSQ rating when deterioration (or improvement) was perceived.

**Table 2:** Mean change (95% confidence interval) and scale interpretation for QLQ-C30 scores after treatment start

**Clinical service**		**QLQ-C30 domain**				
		**Physical function**	**Emotional function**	**Social function**	**Cognitive function**	**Global QOL**
**SCT**	**Change**	−15.80 (−18.44,-13.17)	1.86 (−0.40, 4.13)	−11.76 (−16.26, -7.26)	−9.51 (−12.62, -6.40)	−19.19 (−22.26, -16.12)
	Scale*	Medium	Trivial	Medium	Medium	Large
**MED/RAD**	**Change**	−4.10 (−5.54, -2.67)	3.15 (1.56, 4.74)	−2.91 (−5.27, -0.55)	−1.46 (−3.11,-0.20)	−3.40 (−5.11, -1.69)
	Scale*	Trivial	Trivial	Trivial	Small	Trivial

*Scale interpretation is based on guideline recommended by Cocks et al [13].

The SSQ rating categories were scored from −3 (very much worse) to 3 (very much better) with 0 indicating “about the same.” As suggested by Osoba et al [5], a linear trend between QLQ-C30 change score and the SSQ rating was indication that the magnitude of QLQ-C30 score reflected the degree of change experienced by groups of patients. In this study we defined the MCID as the mean difference in QLQ-C30 score changes that reflected one category change measured by SSQ rating using linear regression. A two-piece linear model was used allowing for different slopes of improvement and deterioration in HRQOL, but was constrained by using the same intercept. Therefore, by definition, the slopes represent MCIDs and the intercept was the mean QLQ-C30 score change among patients perceiving “about the same” in the SSQ.

## Results

### Sample

Table 1 displays demographic and clinical characteristics for the SCT and MED/RAD groups. As illustrated in Table 1, most study participants in both patients groups were white, married/partnered and had some college education.

### QLQ-C30 scores and SSQ ratings

The mean QLQ-C30 scores were significantly lower at T2 than T1 for PF, SF, CF and the global QOL domains for both MED/RAD and SCT patients, while higher at T2 for EF among both patient groups. Table 2 lists the mean change (95% CI) in QLQ-C30 scores for each domain and global QOL. Based on the guidelines on longitudinal QOL change recommended by Cocks et al. [13], the decrease is considered medium in PF (−15.8), SF (−11.76), and CF (−9.51), large in global QOL (−19.19) among SCT patients; and is considered trivial in PF (−4.10), SF(−2.91), CF (−1.46) and global QOL (−3.40) among MED/RAD patients. On the other hand, the mean EF score increases observed in this study were considered trivial for SCT patients (1.86) and MED/RAD patients (3.15).

From the SSQ ratings of most subscales (Figure 1), more patients reported “about the same” than other response. More SCT patients perceived deteriorated HRQOL than improvement while on treatment; for example, 52% verse 16% on global QOL. MED/RAD patients perceived rates of improvement similar to those of deterioration; for example, 25% versus 26% on global QOL.

### Association of QLQ-C30 score changes and SSQ ratings

Overall, the correlations between the QLQ-C30 score change and the SSQ rating categories ranged from 0.28 (SF) to 0.40 (global QOL) among MED/RAD patients, and from 0.25 (SF) to 0.40 (CF) among SCT patients. For patients who responded “about the same” on the SSQ, the mean change in QLQ-C30 for the global QOL domain deteriorated 14.2 and 2.0 units for SCT and MED/RAD patients, respectively (Figure 2). The direction in the mean QLQ-C30 change scores were aligned with the perceived change reported on the SSQ among MED/RAD patients; the mean QLQ-C30 score changes increased, from negative to positive, as corresponding SSQ ratings indicating better perceived change. This pattern was only observed among SCT patients reporting deteriorated HRQOL. Among SCT patients with improvement on the SSQ, the mean QLQ-C30 change scores were negative in most instances, indicating a deteriorating QLQ-C30 score at T2.

Effect sizes of 0.2, 0.5 and > 0.8 reflect small, moderate, and large changes, respectively, according to Cohen [19]. Effect sizes for the “about the same” SSQ response were larger than 0.2 in the global QOL (−0.77), PF (−0.40) and EF (0.26) among SCT patients, and for EF (0.31) among MED/RAD patients (Table 3). Effect sizes were moderate to large (≥ 0.5) in 14/15 (SCT) and 12/15 (MED/RADF) instances when deterioration perceived on the SSQ ratings. Among patients who perceived improvement, only small effect sizes (0.2 to 0.5) in the same direction as the SSQ ratings were observed in 6/15 (SCT) and 8/15(MED/RAD) instances.

**Table 3:** Effect sizes and standard deviations for QLQ-C30 change scores with the corresponding SSQ categories

**QLQ-C30 domain**	**Clinical service**	**SSQ rating categories**						
		**Very much worse**	**Moderately worse**	**A little worse**	**About the same**	**A little better**	**Moderately better**	**Very much better**
**Physical function**	**SCT**	−2.1	−0.97	−1.2	−0.4	−1.17	−0.17	0.04
		(12.2)	(20.2)	(15.0)	(13.0)	(17.5)	(23.9)	(22.5)
	**MED/RAD**	−1.38	−0.94	−0.42	−0.17	−0.07	−0.02	0.17
		(16.5)	(18.6)	(15.5)	(9.5)	(16.4)	(19.5)	(17.9)
**Emotional function**	**SCT**	NA	−0.76	−0.03	0.26	0.27	0.63	0.47
			(13.9)	(13.9)	(15.5)	(13.7)	(23.0)	(20.1)
	**MED/RAD**	−0.86	−0.73	−0.25	0.31	0.44	0.38	0.65
		(23.2)	(22.9)	(16.4)	(13.0)	(15.1)	(21.6)	(20.4)
**Social function**	**SCT**	−0.69	−0.63	−0.78	−0.08	−0.31	−0.21	0.3
		(31.5)	(32.2)	(25.6)	(30.2)	(27.1)	(39.7)	(56.1)
	**MED/RAD**	−0.72	−1.18	−0.4	0.02	−0.08	0.16	0.42
		(33.4)	(22.6)	(24.7)	(21.3)	(29.0)	(22.2)	(33.8)
**Cognitive function**	**SCT**	−1.61	−0.79	−1.02	−0.06	0.51	−0.73	−0.29
		(28.5)	(23.9)	(16.0)	(20.4)	(19.6)	(13.1)	(19.2)
	**MED/RAD**	−0.48	−0.78	−0.51	0.06	0.43	0.13	0.73
		(43.9)	(32.6)	(18.3)	(12.5)	(21.6)	(16.5)	(19.5)
**Global QOL**	**SCT**	−1.28	−1.43	−1.28	−0.77	−0.8	0.26	0.17
		(28.2)	(20.8)	(17.7)	(18.4)	(17.0)	(27.0)	(25.0)
	**MED/RAD**	−0.77	−1.31	−0.76	−0.13	0.22	0.54	0.17
		(43.3)	(20.9)	(15.9)	(15.4)	(16.0)	(16.3)	(20.5)

For patients who perceived “about the same” in SSQ, we identified a significant change in QLQ-C30 scores for PF (−9.2), EF (4.1) and global QOL (−15.2) among SCT patients, and only for EF (4.0) among MED/RAD patients (Table 4). A linear trend between QLQ-C30 score changes and the corresponding SSQ ratings was observed when perceived deterioration in HRQOL was reported; thus, the defined MCID ranged from 5.7 to 11.4 among SCT patients, and from 7.2 to 11.8 among MED/RAD patients (Table 4). For example, in the PF domain, one category improvement on perceived change in the SSQ rating (e.g., from very much worse to moderately worse), was associated, on average, with a 5.7 unit increase in the QLQ-C30 score change among SCT patients and 7.2 unit increase among the MED/RAD patients. For the global QOL domain, the increase in the QLQ-C30 score changes associated with one category improvement in the SSQ rating were 7.3 (SCT) and 11.8 (MED/RAD) units. The MCID among MED/RAD patients for perceived improvement was small (2.7 to 3.3). Excepting for the global QOL domain (estimate = 6.9), no linear relationship between the QLQ-C30 change score and the SSQ ratings was observed for perceived improvement among SCT patients; therefore, no meaningful difference was detected.

**Table 4:** The relationship between the EORTC QLQ-C30 change scores and the SSQ rating categories during cancer treatment

**QLQ-C30 Domain**	**Clinical service**	**Intercept**		**Slope-improvement**		**Slope-deterioration**	
		**Est.**	**p-value**	**Est.**	**p-value**	**Est.**	**p-value**
**Physical function**	**SCT**	−9.21	0	1.72	0.36	5.70	<.0001
	**MED/RAD**	−1.30	0.19	1.11	0.14	7.15	<.0001
**Emotional function**	**SCT**	4.09	0.0074	2.22	0.17	6.35	.0005
	**MED/RAD**	4.04	0	2.68	0.002	9.12	<.0001
**Social function**	**SCT**	−5.75	0.073	3.39	0.41	7.71	.002
	**MED/RAD**	−0.35	0.82	2.95	0.042	10.60	<.0001
**Cognitive function**	**SCT**	−1.33	0.51	−0.61	0.82	11.39	<.0001
	**MED/RAD**	0.89	0.35	3.21	0.005	10.79	<.0001
**Global QOL**	**SCT**	−15.22	0	6.85	0.005	7.27	<.0001
	**MED/RAD**	−1.37	0.20	3.33	0.0007	11.76	<.0001

## Discussion

In a large sample of patients with various cancer types treated at two comprehensive cancer centers, our results reveal several important observations. First, the SSQ was a feasible metric with which to conduct an anchor-based analysis of associations between perceived change and self-reported HRQOL change. Second, modest correlations were found between QLQ-C30 and SSQ, with most domain scores reflected worse QOL during active therapy as compared to pre-treatment. Third, and perhaps most notable, was the large discrepancy of the scoring of diminished physical function and global QOL on the QLQ-C30 among SCT patients who concurrently perceived no change or improvement in the corresponding SSQ items. However, about 50% patients reported “about the same” on the SSQ for most domains, indicating a perceived stability of HRQOL throughout treatment. Finally, we found differential MCID estimates among different domains as well as for improved HRQOL versus deteriorated HRQOL.

Our findings suggest cancer treatment has a negative impact on HRQOL among patients with cancer, and that impact is greater among transplant patients. Based on recent guidelines [13], the deterioration is regarded as medium to large among transplant patients for most domains. These findings support the universal understanding that cancer therapy results in multiple side effects and interferes with nearly all aspects of life. We observed that PF, SF and CF as well as global QOL deteriorated, while EF improved over time. This pattern has been documented in other longitudinal studies using the QLQ-C30 ; domain scores related to physical function diminished from pre-treatment to on- or immediately after treatment and emotional function improved [20]. The initial anxiety of the diagnosis and treatment initiation period may have been ameliorated by subsequent familiarity and supportive psychosocial care provided by the clinical service teams. The greater magnitude of HRQOL deterioration among SCT patients at T2 (immediately after hospitalization) is supported by Grulke et al.’s findings that HRQOL is lowest while in the hospital, but returns to pre-transplant level one year after transplant [7].

In line with Cohen’s operational definition [19], both Osoba et al [5] and Cocks et al [8] have recommended thresholds of trivial (<0.2), small (0.2-0.5) and large (>0.5) effect sizes. However, we observed a larger magnitude of effect sizes when “worse” was perceived on the SSQ ratings. For example, most effect sizes were larger than 0.5 even when “a little worse” was reported. This is not surprising, as King previously reported that change in HRQOL observed before and during cancer treatment is often larger than those observed between two treatment arms [6]. We also observed large standard deviations for the majority of instances, indicating diversity in patients’ health conditions and perceived changes during treatment. On the other hand, the effect sizes did not vary substantially between the rating of “a little” and “a lot” among the instances. Given these phenomena, the interpretation of difference in a given study requires more consideration and research.

The MCID from our study ranged 5.7-11.4 (SCT) and 7.2-11.8 (MED/RAD) among patients reporting deteriorated HRQOL; and 2.7-3.4 among MED/RAD patients reporting improvement, which are in similar magnitude as previously reported among European patients, with 6–12 points for breast cancer patients [5], and 5–14 points for brain cancer patients [11]. Compared with the recent guidelines by Cocks et al [13] the range of MCID is in line with the thresholds for “small” changes which were referred as “subtle but nevertheless clinical relevant changes”. Thus, the findings are in agreement from the two studies. Consistent with previous findings [5,6,8,11,13], we observed the MCID of QLQ-C30 varied across domains and among different patient populations. King and colleagues [6] also reported different magnitudes in MCID among different patient groups, supporting differences in MCID between MED/RAD and SCT patients in our study.

Our results suggest a larger MCID was related to deterioration versus improvement among both MED/RAD and SCT patients. This phenomenon was previously observed in other QOL instrument (FACT) [10], as well as in the QLQ-C30 [12]. In a recent meta-analysis on 118 longitudinal studies, Cocks et al. observed smaller estimates for improvement than for deterioration [13]. Considering these available findings, it appears that patients may be more sensitive to favorable differences, thus, a smaller MCID should be used to interpret QOL improvement.

We also observed decreases in QLQ-C30 scores among patients reporting “about the same” in the SSQ for the PF and global QOL domains. This finding suggests a potential response shift in scoring of HRQOL. Response shift is known as the change in internal standards, values, and the conceptualization of HRQOL after the start of cancer treatment [21]. Patients may report a better health condition even though their actual physical condition has deteriorated when they perceive a greater survival benefit from cancer treatment [21,22]. Further exploration and conceptual work are necessary to better understand the effect of response shift on patient-reported HRQOL scores.

The generalizability of our findings to other samples may be limited; our sample was relatively homogenous in terms of race and education and was limited to patients treated at a comprehensive cancer center, and the time interval under study was specific to before and during cancer treatment. Only one anchor (SSQ) was included in the study and its stability has some discussion [23], thus the robustness of the identified MCID was checked by comparing with previous reports in the literature.

## Conclusions

We conducted a systematic and comprehensive evaluation of change in HRQOL before and during treatment in a large sample of American patients with cancer. Our findings may provide relevant information for managing cancer patients’ HRQOL during active therapy. Our study suggests different MCID thresholds should be applied to interpret QLQ-C30 change from pre-treatment to during/post treatment among domains and between improved and deteriorated HRQOL. Specifically, clinical attention can be focused on patients who report at least a 6 point decrease, and for patients who report at least a 3 point increase on QLQ C-30 domains.

## Consent

Written informed consent was obtained from the patient for the publication of this report and any accompanying images.

## Abbreviations

HRQOL: Health related quality of life; MCID: Minimal clinical important difference; QLQ-C30: Quality of Life Questionnaire-Core 30; SCT: Stem cell transplant; MED: Medical oncology; RAD: Radiation oncology; PF: Physical function; EFV: Emotional function; SF: Social function; CF: Cognitive function; SSQ: Subject significance questionnaire; QOL: Quality of life.

## Competing interests

There are no competing interests to declare.

## Authors’ contribution

F Hong: statistical analysis, manuscript preparation and coordinator; JL Bosco: discussion, manuscript review and editing; N Bush: original idea, manuscript review and editing; DL Berry: design and development of the study, manuscript review and editing and coordinator. All authors read and approved the final manuscript.

## Pre-publication history

The pre-publication history for this paper can be accessed here:

http://www.biomedcentral.com/1471-2407/13/165/prepub

## References

[B1] Osthus AA Aarstad AK Olofsson J Health-related quality of life scores in long-term head and neck cancer survivors predict subsequent survival: a prospective cohort study Clin Otolaryngol 2011 36 361 8 10.1111/j.1749-4486.2011.02342.x 21624101

[B2] Svensson H Hatschek T Johansson H Health-related quality of life as prognostic factor for response, progression-free survival, and survival in women with metastatic breast cancer 2011 Med Oncol 10.1007/s12032-011-9844-9 21298494

[B3] Wan Leung S Lee TF Chien CY Health-related quality of life in 640 head and neck cancer survivors after radiotherapy using EORTC QLQ-C30 and QLQ-H&N35 questionnaires BMC Cancer 2011 11 128 10.1186/1471-2407-11-128 21486431 PMC3083374

[B4] Aaronson NK Ahmedzai S Bergman B The European Organization for Research and Treatment of Cancer QLQ-C30: a quality-of-life instrument for use in international clinical trials in oncology J Natl Cancer Inst 1993 85 365 76 10.1093/jnci/85.5.365 8433390

[B5] Osoba D Rodrigues G Myles J Interpreting the significance of changes in health-related quality-of-life scores J Clin Oncol 1998 16 139 44 9440735 10.1200/JCO.1998.16.1.139

[B6] King MT The interpretation of scores from the EORTC quality of life questionnaire QLQ-C30 Qual Life Res 1996 5 555 67 10.1007/BF00439229 8993101

[B7] Grulke N Albani C Bailer H Quality of life in patients before and after haematopoietic stem cell transplantation measured with the European Organization for Research and Treatment of Cancer (EORTC) Quality of Life Core Questionnaire QLQ-C30 Bone Marrow Transplant 2012 47 473 82 10.1038/bmt.2011.107 21602898

[B8] Cocks K King MT Velikova G Evidence-based guidelines for determination of sample size and interpretation of the European Organisation for the Research and Treatment of Cancer Quality of Life Questionnaire Core 30 J Clin Oncol 2011 29 89 96 10.1200/JCO.2010.28.0107 21098316

[B9] Ringash J O’Sullivan B Bezjak A Interpreting clinically significant changes in patient-reported outcomes Cancer 2007 110 196 202 10.1002/cncr.22799 17546575

[B10] Cella D Hahn EA Dineen K Meaningful change in cancer-specific quality of life scores: differences between improvement and worsening Qual Life Res 2002 11 207 21 10.1023/A:1015276414526 12074259

[B11] Maringwa J Quinten C King M Minimal clinically meaningful differences for the EORTC QLQ-C30 and EORTC QLQ-BN20 scales in brain cancer patients Ann Oncol 2011 22 2107 12 10.1093/annonc/mdq726 21324954

[B12] Kvam AK Fayers PM Wisloff F Responsiveness and minimal important score differences in quality-of-life questionnaires: a comparison of the EORTC QLQ-C30 cancer-specific questionnaire to the generic utility questionnaires EQ-5D and 15D in patients with multiple myeloma Eur J Haematol 2011 87 330 7 10.1111/j.1600-0609.2011.01665.x 21668504

[B13] Cocks K King MT Velikova G Evidence-based guidelines for interpreting change scores for the European Organisation for the Research and Treatment of Cancer Quality of Life Questionnaire Core 30 Eur J Cancer 2012 48 11 1713 1721 10.1016/j.ejca.2012.02.059 22418017

[B14] Jaeschke R Singer J Guyatt GH Measurement of health status. Ascertaining the minimal clinically important difference Control Clin Trials 1989 10 407 15 10.1016/0197-2456(89)90005-6 2691207

[B15] Osoba D Interpreting the meaningfulness of changes in health-related quality of life scores: lessons from studies in adults Int J Cancer Suppl 1999 12 132 7 10679884 10.1002/(sici)1097-0215(1999)83:12+<132::aid-ijc23>3.0.co;2-4

[B16] Berry DL Blumenstein BA Halpenny B Enhancing patient-provider communication with the electronic self-report assessment for cancer: a randomized trial J Clin Oncol 2011 29 1029 35 10.1200/JCO.2010.30.3909 21282548 PMC3068053

[B17] Fayers P Aaronson N Bjordal K EORTC QLQ-C30 Scoring Manual (ed 3rd) 2001 EORTC: Brussels

[B18] Rodrigues G Bezjak A Osoba D The relationship of changes in EORTC QLQ-C30 scores to ratings on the Subjective Significance Questionnaire in men with localized prostate cancer Qual Life Res 2004 13 1235 46 15473502 10.1023/B:QURE.0000037494.27127.b5

[B19] Cohen J Statistical power analysis for the behavioral sciences 1988 Hillsdale: Erlbaum

[B20] Frodin U Borjeson S Lyth J A prospective evaluation of patients’ health-related quality of life during auto-SCT: a 3-year follow-up Bone Marrow Transplant 2011 46 1345 52 10.1038/bmt.2010.304 21113189

[B21] Schwartz CE Sprangers MA Methodological approaches for assessing response shift in longitudinal health-related quality-of-life research Soc Sci Med 1999 48 1531 48 10.1016/S0277-9536(99)00047-7 10400255

[B22] Tierney DK Facione N Padilla G Response shift: a theoretical exploration of quality of life following hematopoietic cell transplantation Cancer Nurs 2007 30 125 38 10.1097/01.NCC.0000265002.79687.af 17413778

[B23] Kamper SJ Maher CG Mackay G Global rating of change scales: a review of strengths and weaknesses and considerations for design The Journal of manual & manipulative therapy 2009 17 3 163 170 20046623 10.1179/jmt.2009.17.3.163 PMC2762832

